# Derivation of transgene-free bat induced pluripotent stem cells amenable to chimera formation in mice, pigs, and chicks

**DOI:** 10.1038/s41421-023-00587-3

**Published:** 2023-09-05

**Authors:** Yumin Qin, Chongyang Li, Xin Gao, Yuanyuan Wu, Zihang Guo, Fei Gao, Dawei Yu, Sen Wu, Xuguang Du

**Affiliations:** 1 State Key Laboratory of Animal Biotech Breeding, College of Biological Sciences, China Agricultural University, Beijing, China; 2 State Key Laboratory of Animal Biotech Breeding, Institute of Animal Science, Chinese Academy of Agricultural Sciences, Beijing, China; 3 National Germplasm Center of Domestic Animal Resources, Institute of Animal Science, Chinese Academy of Agricultural Sciences, Beijing, China; 4 Sanya Institute, China Agricultural University, Sanya, Hainan China

**Keywords:** Reprogramming, Pluripotent stem cells

Dear Editor,

The unique biological characteristics of bats, including longevity, antiviral properties, hibernation, and echolocation, have garnered significant attention from the scientific community. To enhance the utilization of bats in biological experimentation, researchers have undertaken efforts to generate induced pluripotent stem cells (iPSCs) from bat fibroblasts^1,2^. However, it remains uncertain whether transgene-free iPSCs can differentiate in vivo, which indicates a high degree of stem cell quality and greatly expands their potential for various applications.

**Fig. 1:**
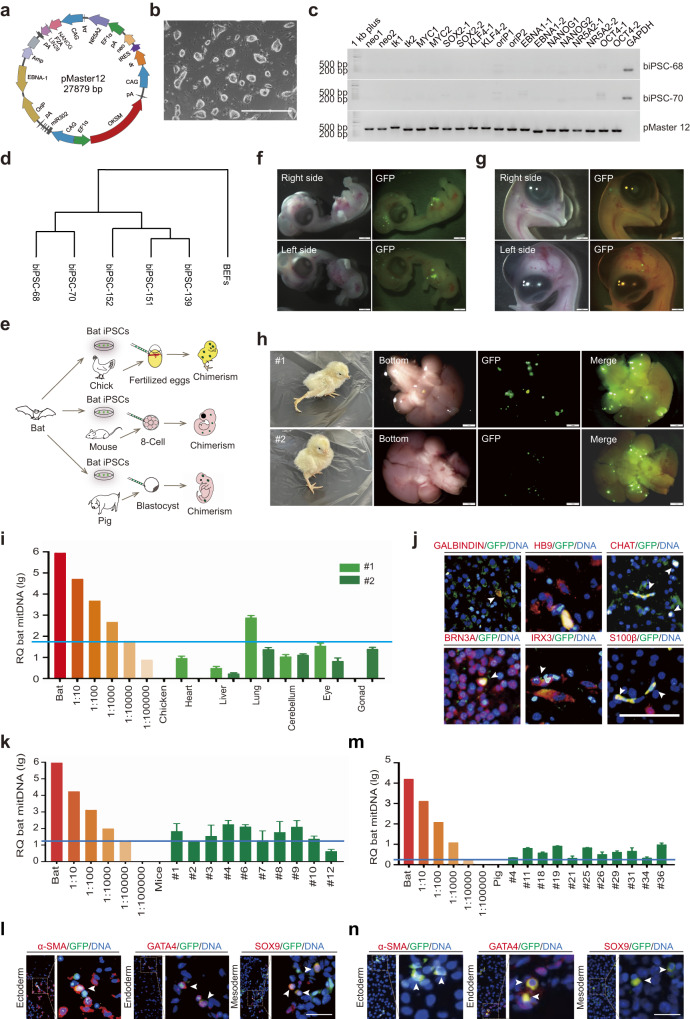
Generation of transgene-free biPSCs capable of in vivo differentiation in mice, pigs, and chicks. **a** Schematic diagram of the inducible vector, pMaster12, encoding 8 reprogramming factors with positive/negative selection genes. **b** Image of biPSCs; scale bar, 1000 µm. **c** PCR analysis confirmed the absence of pMaster12 vector sequences in biPSC-68 and biPSC-70. *GAPDH* was used as the internal reference gene, and purified pMaster12 DNA served as the positive control. **d** Dendrogram clustering of gene expression profiles: BEFs, transgene, and transgene-free biPSCs. **e** Illustrative model: transgene-free biPSCs generate interspecies chimeras (mouse, pig, chick) in vivo. **f** Representative images of D6.5 bat/chick chimeric embryos with GFP fluorescence in the head/chest. Scale bars, 1 mm. **g** Representative images of D9.5 bat/chick chimeric embryos displaying GFP fluorescence in the head. Scale bars, 1 mm. **h** Representative images of interspecies bat/chick live birth chimeras at 3 d post-birth, showing GFP fluorescence in the head. Scale bars, 1 mm. **i** Quantitative genomic PCR analysis of bat mtDNA in chimeric neonatal chicks (#1 and #2). Bat–chick cell dilutions were used for the analysis. The blue line represents the bat mtDNA detection level (1 bat cell per 10,000 chick cells). **j** Representative IF staining images of the head of bat/chick chimera #2, showing positive staining for neural markers (GALBINDIN, HB9, CHAT, BRN3A, IRX3, S100β). White arrows indicate cells positive for both GFP and neural markers. Scale bars, 50 μm. **k** Quantitative genomic PCR analysis of bat mtDNA in tissues of E8.5 chimeric mouse embryos using bat–mouse cell dilutions. The blue line represents the bat mtDNA detection level (1 bat cell per 10,000 mouse cells). **l** Representative IF staining images showing integration of GFP-expressing biPSCs in mouse germ layers. White arrows indicate cells positive for both GFP and lineage markers (α-SMA, GATA4, SOX9). Scale bar, 50 µm. **m** Quantitative genomic PCR analysis of bat mtDNA in E25 chimeric porcine embryos using bat–pig cell dilutions. The blue line represents the bat mtDNA detection level (1 bat cell per 10,000 porcine cells). **n** Representative IF staining images showing integration of GFP-expressing biPSCs in pig germ layers. White arrows indicate cells double positive for GFP and lineage markers (α-SMA, GATA4, SOX9). Scale bar, 50 µm.

To generate transgene-free iPSCs, bat embryonic fibroblasts (BEFs) from *Myotis Lucifugus* were transfected with a set of 8 reprogramming factors (*OCT4*, *SOX2*, *cMYC*, *KLF4*, *NANOG*, *LIN28*, *NR5A2*, and a *miR302*/*367* cluster) in the 3i/LIF medium containing small molecule inhibitors (PD0325901, CHIR99021, and A8301) (Fig. 1a, b). Following positive (neomycin) and negative (thymidine kinase) selection of iPSCs, monoclonal cells were selected for the detection of exogenous factors. We employed 20 sets of primer pairs to amplify the sequence of the plasmid^3^. Results showed that two transgene-free iPSC clones were obtained (Fig. 1c).

Our bat iPSCs (biPSCs) expressed pluripotency markers both at RNA and protein levels (Supplementary Fig. S1a, b). To confirm the pluripotency, we transfected the biPSCs with h*OCT4*-*GFP* and the cells showed bright GFP expression (Supplementary Fig. S1c), indicating the maintenance of pluripotency in biPSCs. The biPSCs exhibited positive alkaline phosphatase (AP) staining and maintained a normal karyotype (42 + XY) after 37 passages (Supplementary Fig. S1d, e). They could be cultured on Matrigel in feeder-free conditions (Supplementary Fig. S2a–c). In vitro, biPSCs could form embryoid bodies and then differentiate into three embryonic germ layers (Supplementary Fig. S3a, b). RNA sequencing analysis revealed distinct clustering of gene expression between the biPSCs and BEFs (Fig. 1d), with high expression of pluripotency genes in biPSCs (Supplementary Fig. S3c). These results suggest that the biPSCs are transgene-free and pluripotent.

To assess the chimerism and developmental capacity of biPSCs, we labeled the biPSCs with GFP/tdTomato (Supplementary Fig. S4a–d) for cell tracking. Chick embryos were selected as a platform for heterologous cell chimerism studies due to the ease of manipulation. We injected 5 × 10^5^ GFP-labeled biPSCs into blood vessels of fertilized chick embryos 52 h after hatching (Fig. 1e). By Day 6.5 (D6.5), significant green fluorescence was observed in the head and chest of the chimeric chick embryos (Fig. 1f), confirmed by GFP staining on paraffin sections (Supplementary Fig. S5a). Co-staining of chimeric chick embryo sections with GFP and germ layer markers confirmed the integration of differentiated biPSCs into all three germ layers (Supplementary Fig. S5b). By D9.5, green fluorescence was limited to the head of chimeric chicks (Fig. 1g).

Two live chimeric chicks were obtained after incubation, and significant GFP fluorescence was observed in their brains (Fig. 1h). To determine the chimeric proportions in other organs, we conducted mitochondrial DNA (mtDNA) analysis. Our results indicated a varying degree of chimerism in different tissues, ranging from 1/100,000 to 1/1000. We detected the integration of green cells in gonads of the chimeric chicks, with one chick showing no chimerism and the other exhibiting less than 1/10,000 chimerism (Fig. 1i). In addition, immunofluorescence (IF) staining for brain paraffin sections (#2) demonstrated differentiation of biPSCs into various neuronal cell types (Fig. 1j). These findings suggest that biPSCs can differentiate into functional neuronal cells in the chimeric chick model.

To examine the chimera-forming potential of biPSCs in two different mammalian models, mice and pigs, we first introduced 10 GFP-labeled biPSCs into mouse embryos at 8–16 cell stage. Among them, 98.09% of injected embryos exhibited significant GFP fluorescence, with an average of 17.78 ± 0.95 biPSCs per blastocyst (Supplementary Fig. S6a–c). biPSCs integrated into the inner cell mass (ICM) of chimeric blastocysts, with an average of 2.93 ± 0.37 cells per ICM (Supplementary Fig. S6d, e). We also assessed the integration of biPSCs into the mouse trophectoderm, revealing their contribution both to the ICM and trophectoderm (Supplementary Fig. S7a). Notably, individual biPSCs exhibited robust proliferation within mouse embryos, with 31.25% of the chimeric embryos displaying single-cell proliferation (Supplementary Fig. S7b and Table S1). Moreover, co-injection of tdTomato-labeled biPSCs and GFP-labeled mouse embryonic stem cells (mESCs) resulted in spatial overlap of red and green fluorescence signals (Supplementary Fig. S7c), suggesting shared development potential between the biPSCs and mESCs within the embryonic microenvironment.

Next, we assessed the chimeric potential of biPSCs in post-implantation mouse embryos (Fig. 1e). We implanted 553 chimeric blastocysts derived from GFP-labeled biPSCs into 33 surrogate mice. Successful pregnancies occurred, and chimeric embryos were collected at embryonic day 8.5 (E8.5). Chimerism was confirmed using genomic PCR with bat-specific primers (Supplementary Fig. S8a), and mtDNA analysis revealed high proportions of chimerism in 8 out of 10 fetuses, with 3 exceeding 1/1000 (Fig. 1k). IF assay at E10.5 showed significant GFP expression in chimeric samples (Supplementary Fig. S8b), with co-expression of GFP and lineage markers indicating differentiation of biPSCs into all three germ layers in the chimeric mouse embryos (Fig. 1l). Our findings demonstrate the remarkable chimeric potential of biPSCs in early mouse embryos, although this potential may diminish as mouse development progresses, and thus we do not achieve neonatal interspecific chimeras.

We then explored the chimeric potential of biPSCs in porcine embryos. GFP-labeled biPSCs were injected into E5 porcine parthenogenic embryos. Among the injected embryos, 92.79% exhibited GFP fluorescence, with an average of 14.14 ± 1.14 biPSCs per blastocyst (Supplementary Fig. S9a–c). IF staining demonstrated the contribution of biPSCs to the ICM, with an average of 3.78 ± 0.31 biPSCs per ICM (Supplementary Fig. S9d, e). These results highlight the viability and integration capability of biPSCs into the ICM of early pig embryos.

We also assessed the chimeric potential of biPSCs in post-implantation porcine embryos. We transplanted 610 in vitro fertilized chimeric blastocysts into 4 surrogate sows. All 4 surrogates became pregnant, resulting in a total of 39 embryos collected between E25–E27. MtDNA genotyping confirmed 11 embryos as chimeras, with biPSCs chimerism exceeding 1/10,000 (Fig. 1m). IF staining with GFP antibody on sections of these chimeric embryos further confirmed the presence of bat cells (Supplementary Fig. S10). IF staining of lineage markers demonstrated the differentiation of biPSCs into all three embryonic germ layers within the chimeric pig embryos (Fig. 1n). These findings suggest that biPSCs exhibit higher chimeric efficiency in early porcine embryos and possess the ability to differentiate into all three embryonic germ layers in vivo.

In this study, we observed successful integration of biPSCs into pre-implantation mouse and pig embryos. However, chimerism observed in post-implantation embryos was limited, which may be influenced by factors such as evolutionary distance, gestation period, and cell competition. Previous studies have shown that rat ESCs and mouse iPSCs were unable to contribute to pig embryos, and the contribution of human PSCs to pig post-implantation embryos was very limited^4^. This limitation may be attributed to the evolutionary distance between species^5^. The evolutionary divergence between bats and mice, estimated to have occurred approximately 94 and 81 million years ago (http://www.timetree.org/), respectively, may significantly affect the efficiency of xenochimerism.

In this study, the highest chimerism ratio and successful generation of live chimeric chicks were observed when biPSCs were injected into chick embryos. The chimeric chicks exhibited differentiation of biPSCs into functional cells in the nervous system, providing valuable insights into biPSCs development and differentiation in heterologous hosts. Chick embryo is a well-established experimental model for xenografts, offering a convenient system for studying embryonic development and differentiation^6^. The chick embryo provides an effective evaluation system for the in vivo developmental potential of xenocells, as previous studies have demonstrated the pluripotency of human PSCs in chick embryos^7^. The increased efficiency of bat/chick chimera may be due to initial higher injected cell numbers and the non-fully developed immune system in chick embryos^8^, which resulted in reduced exogenous cell rejection. Limited gonadal chimerism was due to the injection at a more advanced developmental stage (HH14–HH15) compared to that at the blastocyst stage. Testing different injection stages, sites, and methods in future studies may improve the outcome of chimerism.

Overall, we generated transgene-free biPSCs by electroporation of episomal plasmids. These biPSCs exhibited chimeric potential in three model animals. Transgene-free iPSCs are of higher quality, as they do not have cell lineage differentiation bias and avoid the risk of tumor formation and death in chimeric offsprings^9–11^. They can also differentiate into a broad spectrum of cell types or organoids^12^ under appropriate conditions. Our biPSCs have the potential to differentiate into relevant organoids in vitro, enabling mechanistic investigations of bat longevity, antiviral defense, and echolocation.

### Supplementary information

Supplemental Material File
