# Supplementary material for: Derivation of transgene-free bat induced pluripotent stem cells amenable to chimera formation in mice, pigs, and chicks
Source: Cell Discov. 2023 Sep 5;9:91. doi: 10.1038/s41421-023-00587-3 (PMC10480176; doi:10.1038/s41421-023-00587-3)
Supplement: Supplementary file 1 — Supplemental Material File [file 41421_2023_587_MOESM1_ESM.pdf]

Supplementary Fig. S1

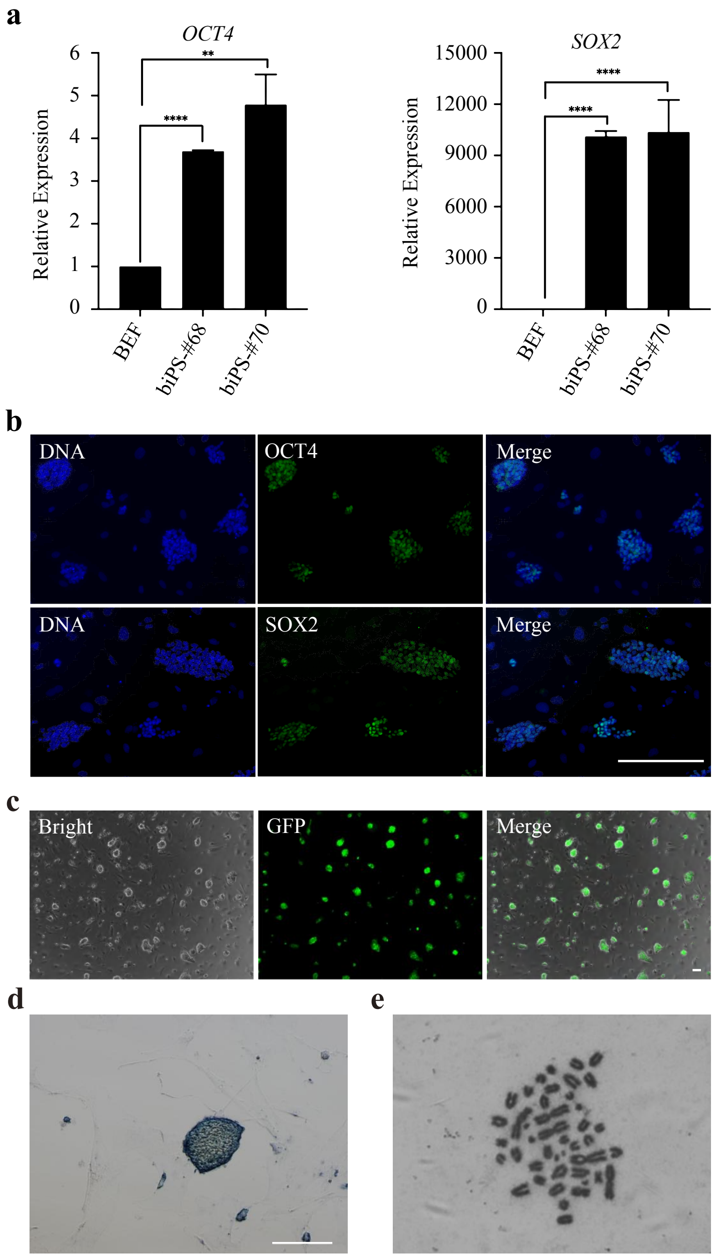

Supplementary Fig. S2

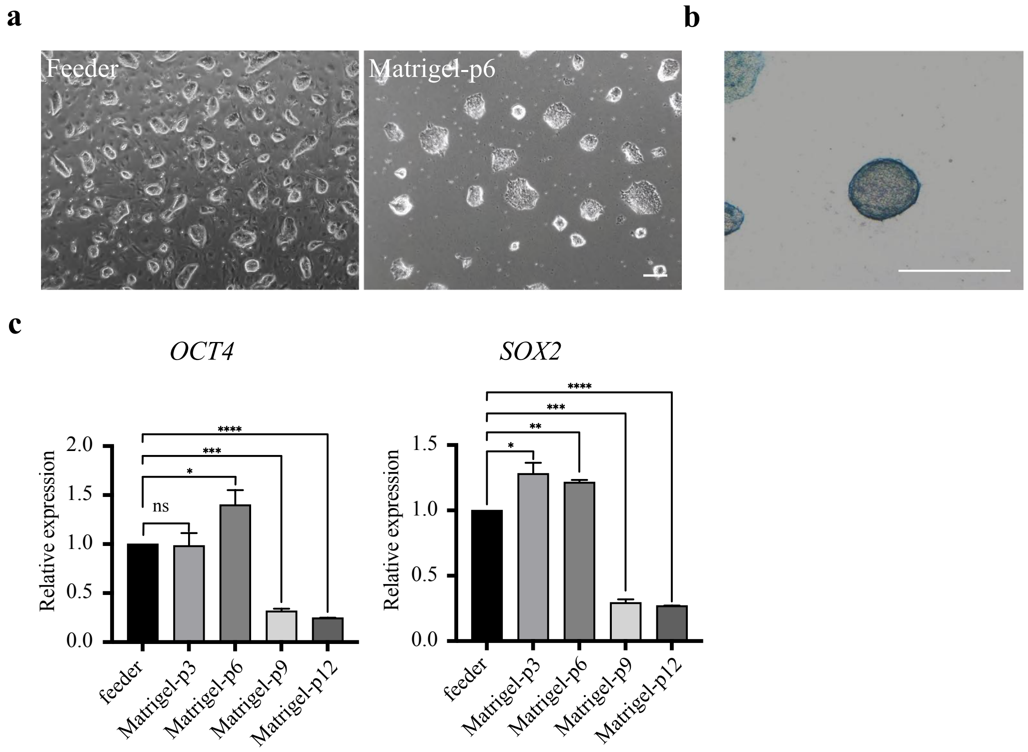

Supplementary Fig. S3

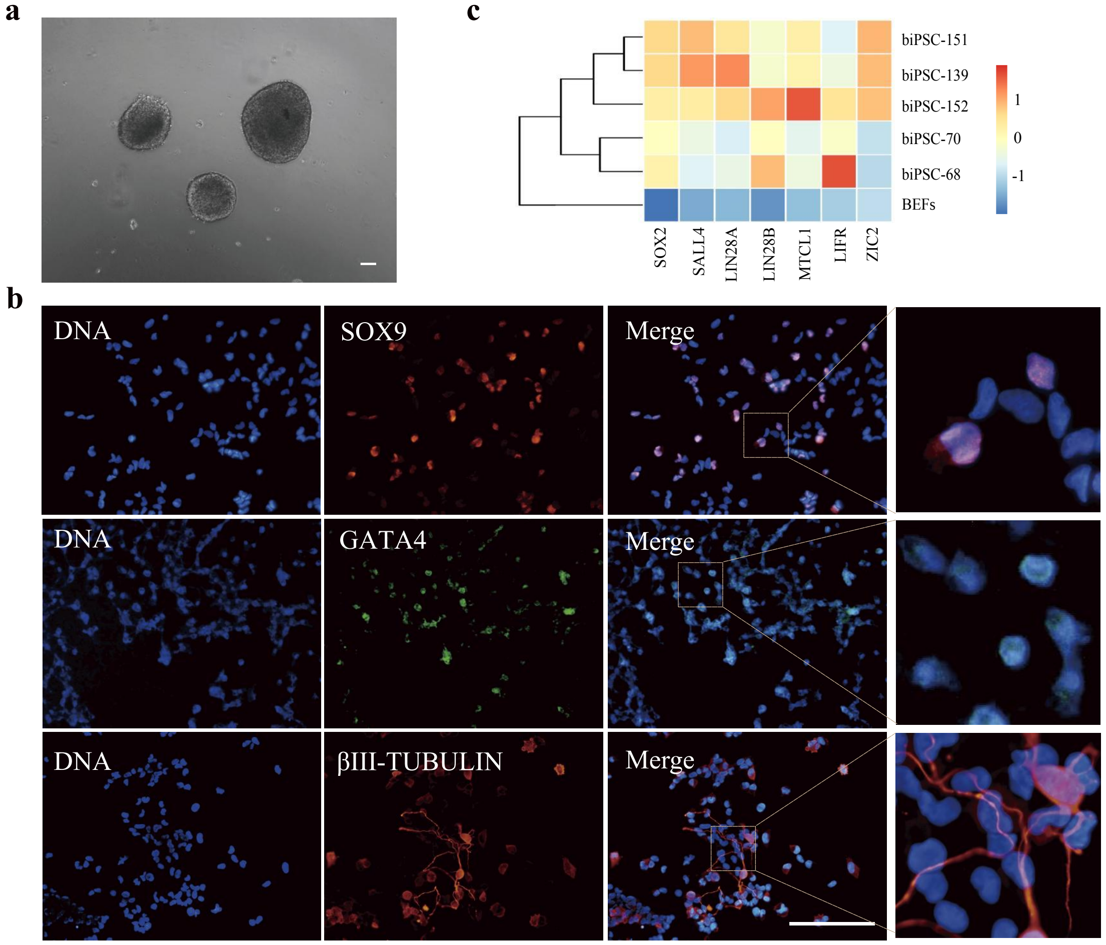

Supplementary Fig. S4

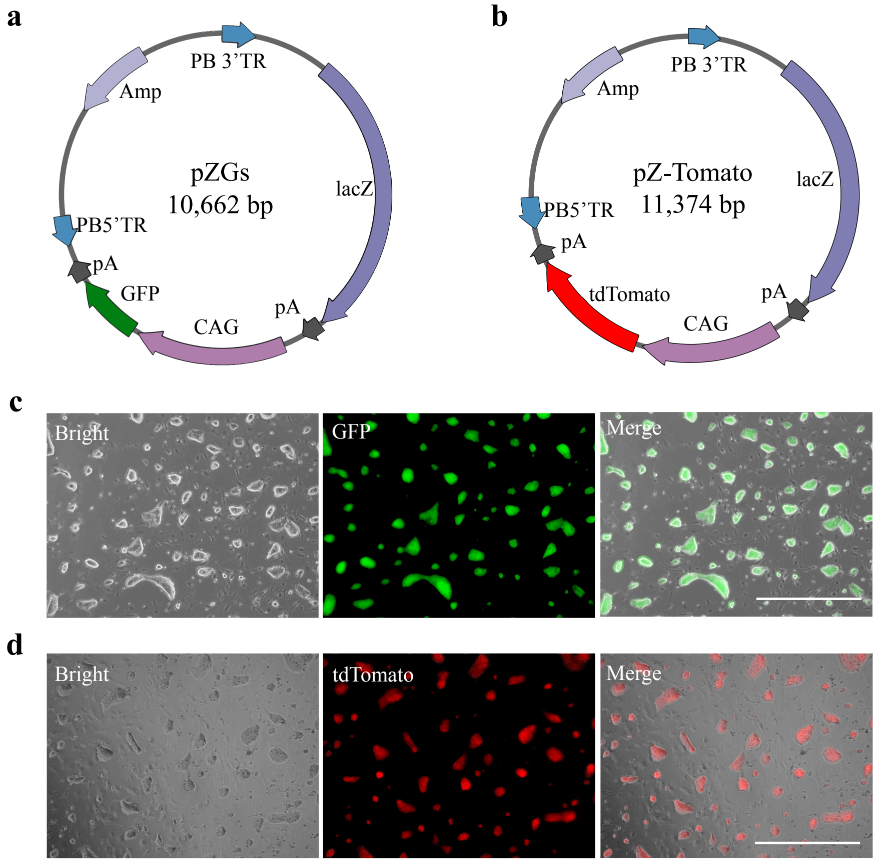

Supplementary Fig. S5

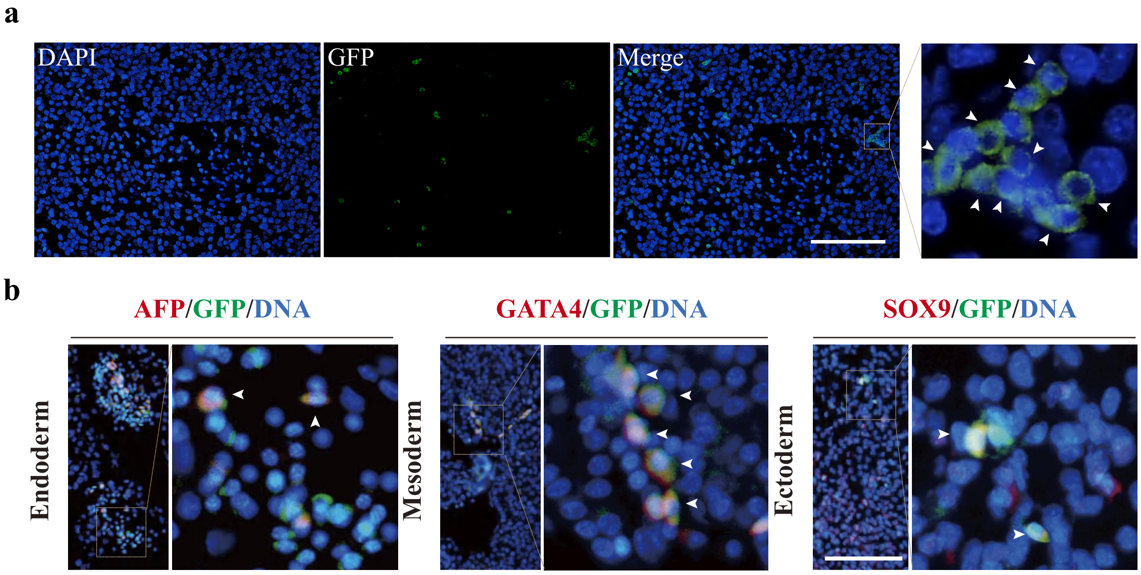

Supplementary Fig. S6

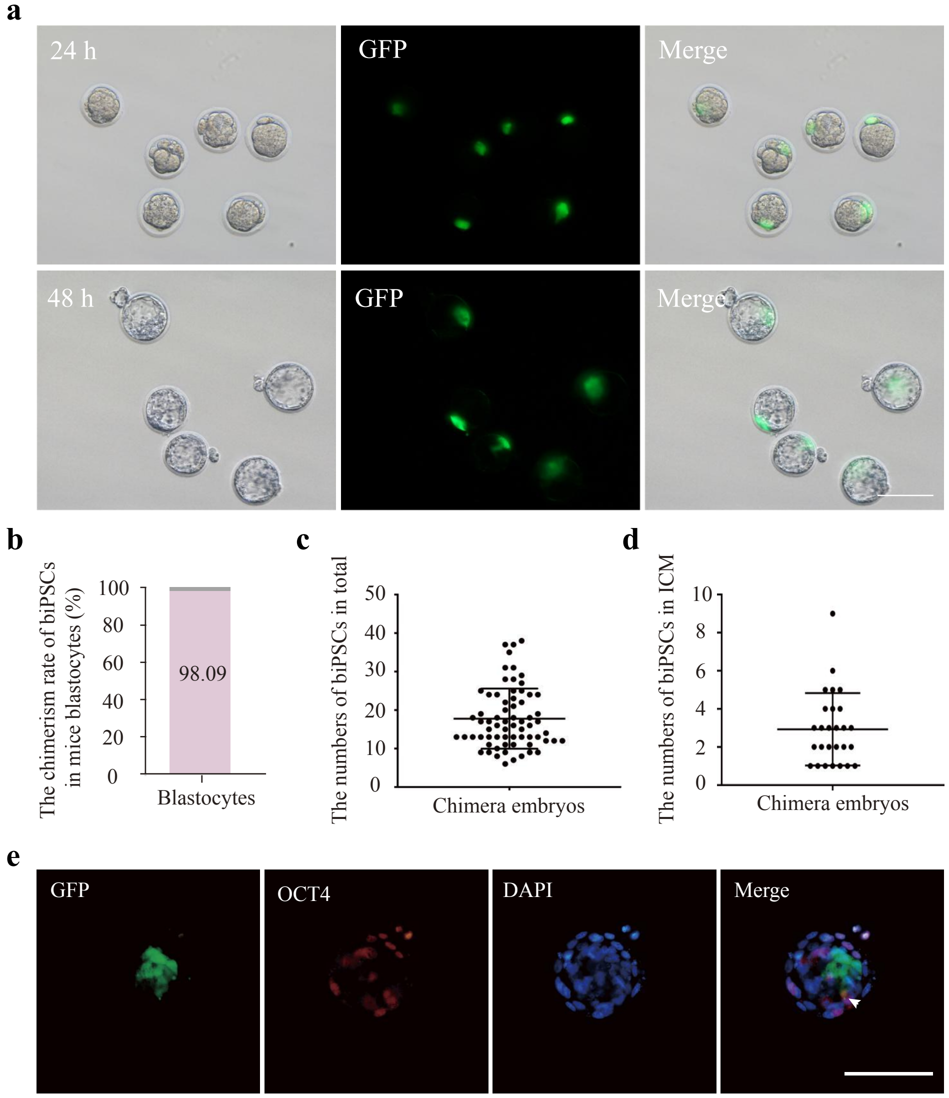

Supplementary Fig. S7

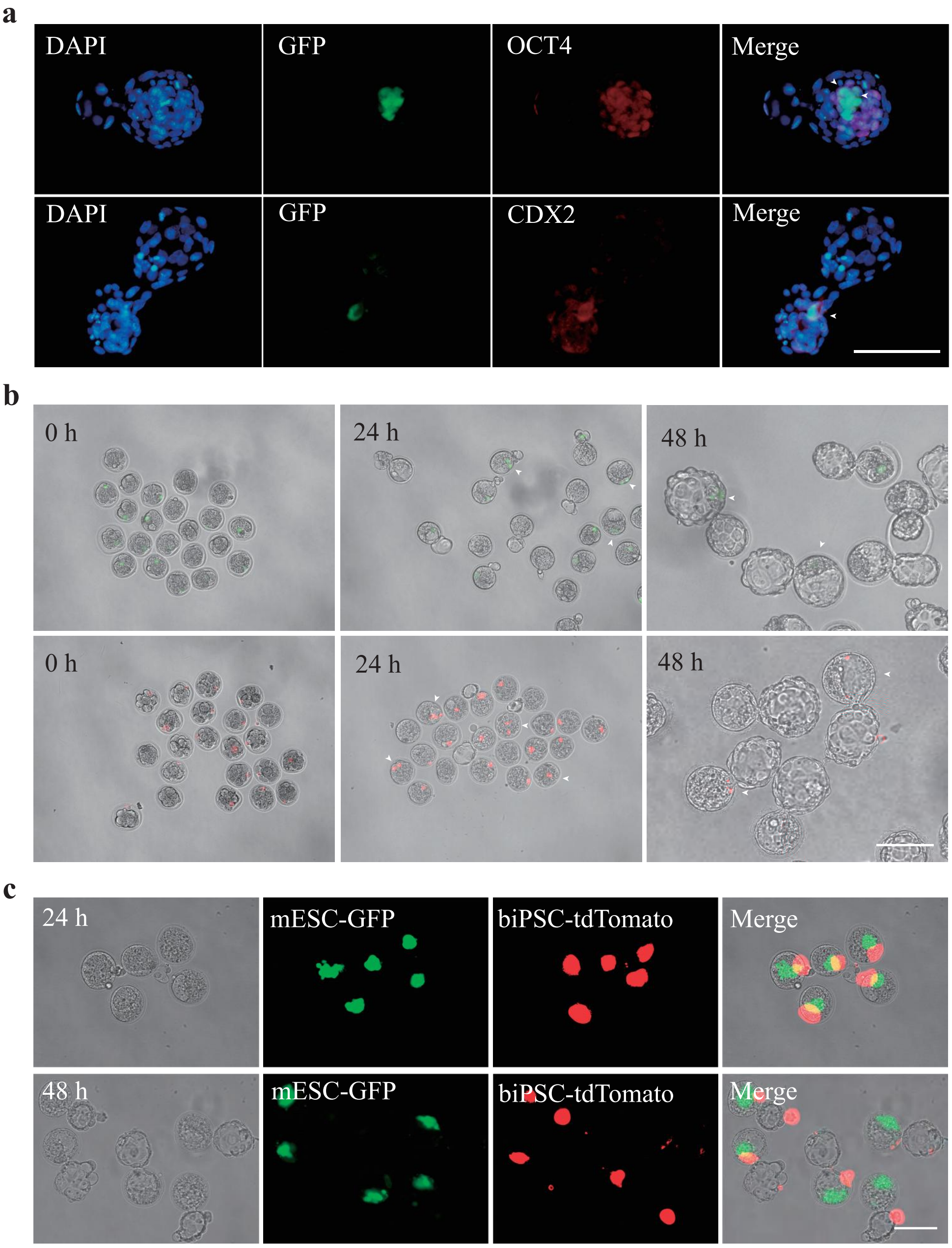

Supplementary Fig.S8

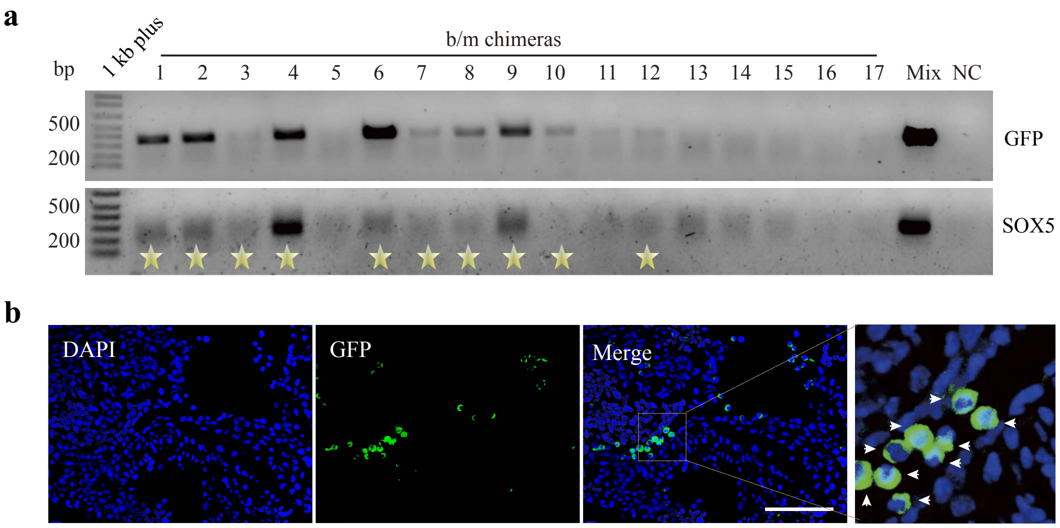

Supplementary Fig. S9

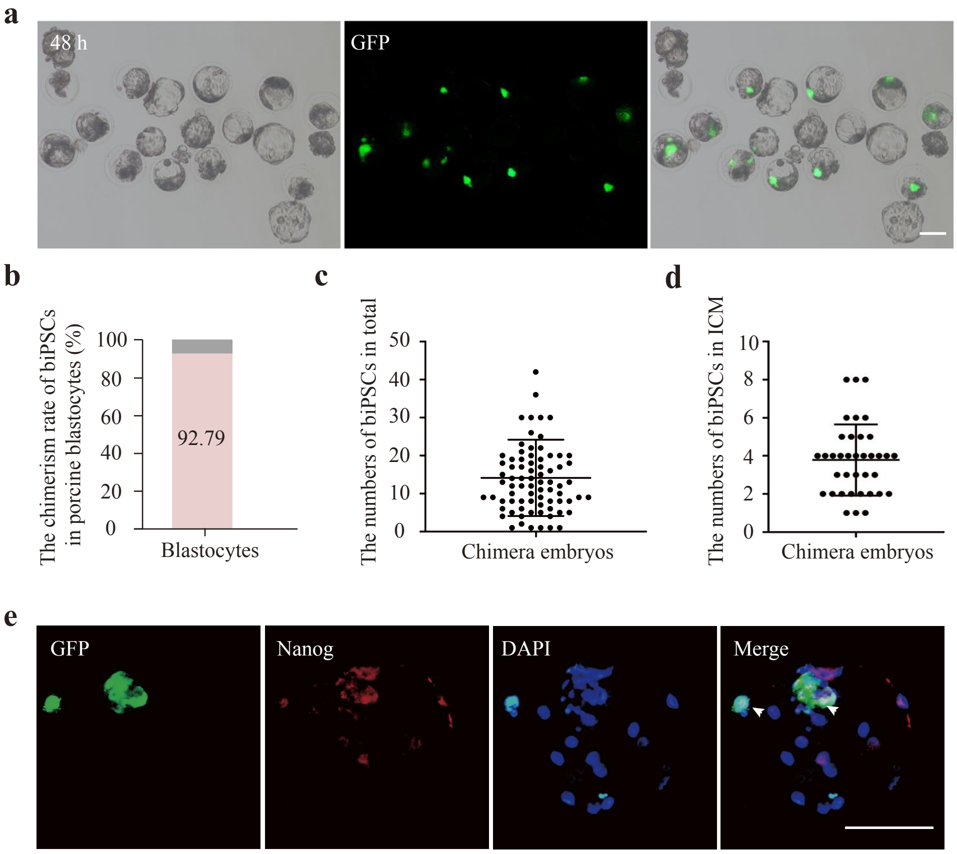

Supplementary Fig. S10

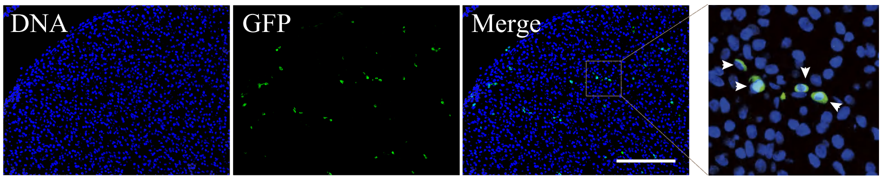

Supplementary Fig. S1 Characterization of biPSCs. a RT-PCR analysis of *OCT3/4* and *SOX2* gene expression in biPSCs, with bat embryonic fibroblast cells (BEFs) as negative controls. β-actin was used as a loading control. Data are represented as Mean ± SD, t test; **$p<0.01$, ****$p<0.0001$. The experiment was repeated 3 times with similar results. **b** IF staining for OCT3/4 and SOX2 in biPSCs. Scale bars, 100 µm. **c** biPSCs showed GFP with human *OCT4* promoter. Scale bars, 100 µm. **d** AP staining after clonal passage. Scale bars, 100 µm. **e** biPSCs maintain a normal karyotype (42+XY) after several passages.

Supplementary Fig. S2 Bat iPSCs cultured on Matrigel in feeder-free condition. a Morphology of biPSCs cultured on feeders and Matrigel. Scale bar, 200 µm. **b** AP staining of biPSCs in Matrigel after eight passages. The image showed the presence of positive AP staining. Scale bar, 200 µm. **c** Expression levels of *OCT4* and *SOX2* in biPSCs cultured in Matrigel. The graph illustrates the changes in gene expression across different passages. Data are represented as Mean ± SD, t test; ns, no significant differences, $p>0.05$, * $p<0.05$, **$p<0.01$, ***$p<0.001$, ****$p<0.0001$. The experiment was repeated 3 times with similar results.

Supplementary Fig. S3 Differentiation capacity of biPSCs. a Morphology of embryoid bodies (EBs) formed from biPSCs. Scale bars, 50 µm. **b** In vitro differentiation ability of biPSCs. IF staining shows expression of lineage markers for the three germ layers in EBs derived from biPSCs, including SOX9 (mesoderm), GATA4 (endoderm), and βIII-TUBULIN (ectoderm). Scale bars, 50 µm. **c** Heat map of pluripotent gene expression in BEFs and biPSCs. biPSC-68 and biPSC-70 are transgene-free, biPSC-139, biPSC-151 and biPSC-152 are transgene iPSCs. The color gradient represents the relative expression levels of each gene, with red indicating higher expression and blue indicating lower expression.

Supplementary Fig. S4 Construction of biPSC with a GFP/tdTomato reporter. a Schematic representation of the fluorescent plasmid vector pZGs, which contains the PB transposase and a GFP element driven by the CAG promoter. **b** Schematic representation of the fluorescent plasmid vector pZ-Tomato, which contains the PB transposase and a tdTomato element driven by the CAG promoter. **c** Representative image of biPSCs displaying green fluorescence, indicative of successful transfection with the pZG plasmid. Scale bars, 1000 µm. **d** Representative image of biPSCs displaying red fluorescence, indicative of successful transfection with the pZ-Tomato plasmid. Scale bars, 1000 µm.

Supplementary Fig. S5 Chimeric competency of biPSCs in chick embryos. a Representative IF staining images of D6.5 chimeric section, demonstrating expression of GFP protein. White arrows indicate cells that are positive for GFP. Scale bar, 50 μm. **b** IF staining of positive chimeric embryos, representative IF images showing the integration of GFP-positive biPSCs and co-expression of lineage markers, including the ectoderm marker AFP, endoderm marker GATA4, and mesoderm marker SOX9. White arrows indicate cells that are positive for both GFP and lineage markers. Scale bar, 50 μm.

Supplementary Fig. S6 Chimeric competency of biPSCs in mice blastocysts. a Representative images of biPSCs development in mouse embryos. biPSCs were labeled with GFP. Scale bars, 100 μm. **b** Proportion of chimeric blastocysts in total mouse blastocysts. **c** The number of integrated biPSCs in total mouse blastocysts. Each dot represents the number of biPSCs in a blastocyst. **d** The number of integrated biPSCs in the mouse blastocysts' ICM. Each dot represents the number of biPSCs in the ICM. **e** Representative images of biPSCs engraftment into the mouse ICM. The injected biPSCs were labeled with GFP, and the ICM marker OCT4 was stained with an anti-OCT4 antibody. Red, OCT4; Blue, DAPI. The white arrows indicate biPSCs contributing to the ICM. Scale bar, 100 μm.

Supplementary Fig. S7 Developmental patterns of biPSCs in mouse embryos. a Representative images illustrating engraftment of GFP-labeled biPSCs in the mouse ICM and trophectoderm. Immunostaining indicates OCT4 expression in the ICM and CDX2 expression in the TE. White arrows indicate biPSCs contributing to mouse ICM and TE development. **b** Injection of single GFP-labeled mESCs and single tdTomato-labeled biPSCs into mouse embryos. Developmental assessment was conducted at 24 hours and 48 hours post-injection. White arrows indicate proliferating mESCs/biPSCs. **c** Representative images of tdTomato-labeled biPSCs and GFP-labeled mESCs in mouse embryos. Scale bar, 100 μm.

Supplementary Fig. S8 Detection of chimerism in mouse post-implantation embryos. a Genomic PCR analysis of E8.5 mice embryos using GFP element and bat specific primers SOX5. 1-17, bat-mouse chimeric embryo samples; Mix, biPSC-GFP/mES (1:1); NC, negative control without genomic DNA. The yellow star indicates samples used for mtDNA detection. Representative gel images are shown. **b** Representative IF staining images of E10.5 chimeric section, demonstrating expression of GFP protein. White arrows indicate cells that are positive for GFP. Scale bar, 50 μm.

Supplementary Fig. S9 Chimeric competency of biPSCs in pig blastocysts. a Representative images of biPSCs development in porcine embryos. biPSCs were labeled with GFP. Scale bars, 100 μm. **b** Proportion of chimeric blastocysts in total porcine blastocysts. **c** The number of integrated biPSCs in total porcine blastocysts. Each dot represents the number of biPSCs in a blastocyst. **d** The number of integrated biPSCs in the porcine blastocysts' ICM. Each dot represents the number of biPSCs in the ICM. **e** Representative images of biPSCs engraftment into the porcine ICM. The injected biPSCs were labeled with GFP, and the ICM marker NANOG was stained with an anti-NANOG antibody. Red, NANOG; Blue, DAPI. The white arrows indicate biPSCs contributing to the ICM. Scale bar, 100μm.

Supplementary Fig. S10 Chimeric competency of biPSCs in pig post-implantation embryos. Representative IF staining images of E25 chimeric section, demonstrating expression of GFP. White arrows indicate cells that are positive for GFP. Scale bar, 50 μm.

Supplementary Table S1 Proliferative capacity of biPSCs within mouse embryos.

**Cell line**	**Embryo count for single-cell injection**	**Embryo count with injected cell proliferation**	**Proliferative rate (%)**
biPSC-tdTomato	32	10	31.25
mESC-GFP	30	13	43.33

Supplementary Table S2 Antibody information

**Antibody**	**Source**	**Cat number**
Mouse Monoclonal Anti-OCT3/4	Santa curz	sc-5279
Mouse Monoclonal Anti-SOX2	Santa curz	365823
Mouse Monoclonal Anti-CDX2	Santa curz	sc-393572
Rabbit polyclonal anti-human NANOG	PeproTech	500-P236
GATA-4 Antibody (G-4) (Ms)	Santa curz	sc-25310
AFP/Alpha fetoprotein Antibody (C3) (Ms)	Santa curz	sc-8399
Rabbit anti SOX9	Cell Signaling	D8G8H
Rabbit anti βIII-TUBULIN	PeproTech	10094
Rabbit anti α-SMA	ABclonal	ab5694
Chick anti GFP	Abcam	ab13970
Rabbit anti BRN3A	Gift from Turner	

Rabbit anti HB9	Gift from Samuel Pfaff	The Salk Institute
Rabbit anti IRX3	Gift from Thomas Jessell	Columbia University
Rabbit anti GALBINDIN	Gift from Swant	
Rabbit anti CHAT	Gift from Chemicon	
Rabbit anti S100β	Abcam	ab52642
Alexa Fluor 488 donkey anti-Mouse IgG H&L	Abcam	ab150105
Alexa Fluor 594 donkey anti-Rabbit IgG H&L	Abcam	ab150108
Alexa Fluor 488 goat anti-Chick IgY H&L	Abcam	ab150173

Supplementary Table S3 PCR primers for genomic PCR and qPCR analysis of *Myotis Lucifugus* mtDNA

**Name**	**Sequences (5'-3')**
YM105-bGAPDH-F	CAACCCTGAGACACGATGGT
YM106-bGAPDH-R	CCGTTCTCAGCCTTGACTGT
YM320-SOX5-F	gcgtgtttaactaaggaaggctatgaat
YM321-SOX5-R	caagacaaagtactacaactggcagaaa
YM142-bmitDNA-F6	cctgagctggcatagtaggcactgcatt
YM143-bmitDNA-R6	gctatatcaggggcgccaattattaaggg
YM237-16SrRNA-F	ccctagggataacagcgcaatc
YM238-16SrRNA-R	ctccggtctgaactcagatcacg

Supplementary Materials and Methods

Ethical approval

All animal experiments were conducted in accordance with the guidelines of Animal Care Committee of China Agricultural University (Approval No: AW03111202-3-1). The cross-species chimeric experiments underwent review and approval by the ethics committee of the China Agricultural University.

Cell culture

The BEFs used in this study were a gift from Mario Capecchi at the University of Utah, USA. They were cultured in Dulbecco's modified Eagle's medium (DMEM; Gibco, 27106) supplemented with 10% fetal bovine serum (FBS; Gibco, Australia, 10099141), 1% L-glutamine, 1% pyruvate, and 1% penicillin streptomycin. The biPSCs were maintained on Co60-irradiation treated mouse embryonic fibroblasts (MEFs) in N2/B27 medium (Gibco, 17502-048/17504-044) supplemented with 1 μM of MEK inhibitor PD0325901 (Selleck, S1036), 3 μM of GSK3 inhibitor CHIR99021 (Selleck, S2924), 1 μM of FGF receptor inhibitor A8301 (Selleck, S7692), and 10 ng/mL of mouse leukemia inhibitory factor (mLIF, Peprotech), a combination referred to as 3i/LIF. The N2B27 medium consisted of a mixture of 500 ml of DMEM/F12 medium (Gibco, 10565-018), 500 ml of Neurobasal medium (Gibco 21103-049), 5 ml of N2 supplement (Gibco 17502-048), and 10 ml of B27 supplement (Gibco 17504-044). The biPSCs were digested into single cells using Tryple (Gibco, 12605-010) and plated onto new wells with MEFs feeder every 2-3 days for further amplification.

Generation of biPSCs

The biPSCs were established following a previously described protocol[1]. Briefly, BEFs were electroporated with pMaster12 plasmid and plated onto new dishes with a MEFs feeder. The following day, the media was changed to ES/LIF (knockout DMEM with 15% FBS and mLIF) with 350 μg/mL G418, and the media was subsequently changed every other day. On day 5 post-electroporation, G418 selection was ceased by changing to medium 3i/LIF. After 9 days, colonies were picked and mechanically dissociated. The dissociated cells (biPSCs) were then plated into new wells (48-well plates with a MEFs feeder) and expanded.

Karyotype analysis

Karyotype analysis was performed as previously described[1]. Briefly, biPSCs were treated with colcemid (0.1 μg/mL, Gibco, 15212-012) for 1 hour. Subsequently, 0.1 mL of fresh fixative solution (3:1, v/v, methanol:acetic acid) was added to the KCl solution, and the mixture was centrifuged at 180 g for 5 minutes. The supernatant was removed, and 5 mL of fixative solution was added, followed by centrifugation at 180 g for 3 minutes. This step was repeated, and the final cell suspension was dropped onto glass slides and stained with Giemsa (Gibco, 10092-013).

Embryoid body formation and differentiation

Embryoid bodies (EBs) were formed by plating biPSCs (1×10^6) in 3i: basic medium = 1:1 in a sterile plastic culture dish. After 48 h, change medium to 3i: basic medium = 3:7, then change medium to totally basic medium 2 days later and continue for 4 days. Aggregates were harvested and maintained in basic medium in new 24-well plate with gelatin (STEMCELL 07903) for further differentiation.

Chick embryo micromanipulation and chimera assays

Chick micromanipulation was performed following published procedures[2,3]. Briefly, fertilized chick embryos (Boehringer Ingelheim) at day 0 were cultured in an incubator at 38°C and approximately 60% relative humidity for 48-52 hours. The eggshell was then opened, and approximately 1 μL of biPSCs suspension (4×10^5) was injected into the marginal vein using a fine glass needle. The eggshell was then closed and returned to the incubator. Chick embryos were collected at 6.5, 9.5 days, and after birth.

Mouse embryo micromanipulation and chimera assays

The procedures for mouse superovulation, zygote collection, embryo culture, and PSCs microinjection were carried out following previously reported protocols[4,5]. Briefly, female ICR-CD1 mice aged 4-6 weeks were superovulated by intraperitoneal injection of 10 IU of equine chorionic gonadotropin (eCG), followed by injection of 10 IU of human chorionic gonadotropin (hCG) 46 hours later. The mice were then mated, and plug-positive females were identified and moved to a fresh cage until E2.5 for harvesting of 8-cell stage embryos. Recipient female ICR-CD1 mice aged 8-12 weeks were mated with male mice (over 8 weeks, ligated, ICR-CD1) at the same time. About 10 GFP-labeled biPSCs were microinjected into the 8-cell stage mouse embryo, which was then cultured overnight until it reached the blastocyst stage. Chimera embryos were collected for IF analysis or embryo transfer. The chimeric embryos (15-18 per unilateral) were transferred into the uteri of 2.5 d pseudo-pregnant mouse foster females.

Porcine embryo micromanipulation and chimera assays

The procedures for porcine oocyte collection, in vitro maturation, in vitro fertilization (IVF), parthenogenetic activation (PA) production, and embryo culture were conducted according to published protocols[6,7]. In brief, Porcine ovaries were collected from a local slaughterhouse and transported to the laboratory within two hours in 38°C normal saline with penicillin-streptomycin added. Porcine oocytes were collected and cultured for 42-44 hours in vitro. After in vitro maturation, the cumulus cells were removed by 0.1% hyaluronidase, and mature oocytes were selected for PA or IVF. The embryos were cultured in PZM3 medium in 5% O2, 5% CO2 at 38.5°C. For in vitro experiments, 10 GFP-labeled biPSCs were microinjected into PA blastocysts cultured for 5 days. After 48 hours of culture, the chimeric embryos were collected for IF analysis. For in vivo experiments, 10 GFP-labeled biPSCs were microinjected into IVF blastocysts. After 24 hours of culture, the chimeric embryos were transferred into recipient surrogates.

Embryos were collected 25 days after pregnancy.

Embryo IF staining

Blastocysts were subjected to IF staining using previously established protocols[8]. Briefly, blastocysts were fixed in 4% paraformaldehyde for 30 minutes at room temperature, followed by permeabilization with 0.5% Triton X-100 in PBS for 1 hour at room temperature. After blocking with 1% bovine serum albumin in PBS for 1 hour at room temperature, primary antibodies (Supplementary Table S2) were applied and incubated overnight at 4°C. The next day, samples were incubated with secondary antibodies for 1 hour at room temperature and stained with 4',6-diamidino-2-phenylindole (DAPI, Beyotime, P0131). Finally, the embryos were imaged using a laser scanning inverted confocal microscope (Nikon, ECLPSE Ti). Throughout the procedure, samples were washed three times before transfer to a new condition.

Genomic PCR

Genomic PCR was performed to detect bat cells in mouse and pig fetuses. Genomic DNA was extracted using established protocols[9], and Taq DNA polymerase was used for PCR. The primer sequences used for PCR are provided in Supplementary Table S3.

Quantitative PCR (qPCR) analysis of *Myotis Lucifugus* mtDNA

Analysis of bat mtDNA was conducted using SYBR Green Realtime PCR Master Mix (Genstar, A311-10). Total DNA was isolated from mice/porcine embryos and neonatal chick tissues. The detection followed previously established procedures[8,10], using a bat mtDNA control and a series of bat/mouse, bat/pig, and bat/chick cell dilutions run in parallel to estimate the degree of bat cells contribution in interspecies chimeras. For normalization, an identical set of reactions was prepared using primers specific for an ultra-conserved non-coding element. The primer sequences used for genomic qPCR are listed in Supplementary Table S3.

Statistical analysis

The statistical analyses were conducted utilizing the Prism 9 Software (GraphPad). To establish statistical significance, the Student's t-test was applied. A P-value less than 0.05 was deemed statistically significant. The data were conveyed as the mean ± standard error (SD).

References

1 Wu, S., Wu, Y., Zhang, X. & Capecchi, M. R. *Proc. Natl. Acad. Sci. USA* **111**, 10678-10683 (2014).

2 Haraguchi, S., Matsubara, Y. & Hosoe, M. *Dev. Growth Differ.* **58**, 194-204 (2016).

3 Jin, K. *et al. Journal of visualized experiments : JoVE* (2022).

4 Okumura, H. *et al. Xenotransplantation* **26**, e12468 (2019).

5 Stepien, B. K., Vaid, S., Naumann, R., Holtz, A. & Huttner, W. B. *STAR protocols* **2**, 100494 (2021).

6 Whitworth, K. M. *et al. Biol. Reprod.* **91**, 78 (2014).

7 Yuan, Y. *et al. Proc. Natl. Acad. Sci. USA* **114**, E5796-e5804 (2017).

8 Fu, R. *et al. Protn & Cell* **11** (2020).

9 Wu, S., Ying, G., Wu, Q. & Capecchi, M. R. *Nat. Protoc.* **3**, 1056-1076 (2008).

10 Hu, Z. *et al. Sci. Adv.* **6**, eaaz0298 (2020).
